# Do Stacked Species Distribution Models Reflect Altitudinal Diversity Patterns?

**DOI:** 10.1371/journal.pone.0032586

**Published:** 2012-03-02

**Authors:** Rubén G. Mateo, Ángel M. Felicísimo, Julien Pottier, Antoine Guisan, Jesús Muñoz

**Affiliations:** 1 Institute of Botany, University of Liège, Liège, Belgium; 2 Real Jardín Botánico (CSIC), Madrid, Spain; 3 Escuela Politécnica, Universidad de Extremadura, Cáceres, Spain; 4 University of Lausanne, Ecology and Evolution Department, Lausanne, Switzerland; Kenya Medical Research Institute - Wellcome Trust Research Programme, Kenya

## Abstract

The objective of this study was to evaluate the performance of stacked species distribution models in predicting the alpha and gamma species diversity patterns of two important plant clades along elevation in the Andes. We modelled the distribution of the species in the *Anthurium* genus (53 species) and the Bromeliaceae family (89 species) using six modelling techniques. We combined all of the predictions for the same species in ensemble models based on two different criteria: the average of the rescaled predictions by all techniques and the average of the best techniques. The rescaled predictions were then reclassified into binary predictions (presence/absence). By stacking either the original predictions or binary predictions for both ensemble procedures, we obtained four different species richness models per taxa. The gamma and alpha diversity per elevation band (500 m) was also computed. To evaluate the prediction abilities for the four predictions of species richness and gamma diversity, the models were compared with the real data along an elevation gradient that was independently compiled by specialists. Finally, we also tested whether our richness models performed better than a null model of altitudinal changes of diversity based on the literature. Stacking of the ensemble prediction of the individual species models generated richness models that proved to be well correlated with the observed alpha diversity richness patterns along elevation and with the gamma diversity derived from the literature. Overall, these models tend to overpredict species richness. The use of the ensemble predictions from the species models built with different techniques seems very promising for modelling of species assemblages. Stacking of the binary models reduced the over-prediction, although more research is needed. The randomisation test proved to be a promising method for testing the performance of the stacked models, but other implementations may still be developed.

## Introduction

Altitudinal gradients have proven to be useful to test general hypotheses on the main drivers shaping global species diversity patterns, such as scale, landscape ecology, and area and season length effects [1]. Altitudinal gradients are complex gradients and thus include variations in several environmental factors, such as temperature, precipitation, topography, erosion, and soil resources [2], which directly influence the growth, persistence and reproduction of organisms [3]. These factors further control the spatial variation in species richness [4]–[14].

The analysis of the biodiversity response to such key ecological gradients mainly involves modelling exercises at community level using comprehensive observational dataset (including a set of environmental variables and data on the distribution of a given organism). Modelling at community level can be performed following different strategies [15], such as direct versus species-assembly approaches [16], each involving different modelling options [17]. The direct strategy of aggregating biological survey data to produce community-level entities that are then modelled (i.e., assemble first, predict later in [15]) has been much used and evaluated, but the alternative strategy of assembling individual species models (i.e., predict first, assemble later in [15]) has been evaluated far less and only more recently [18]–[21], even though many of the assessments of the global threat to biodiversity that have been published were based on such an approach [22]–[24]. This option involves making individual models for all the species included in the analysis separately and then combine them to generate a community level analysis. More specifically, species distribution models (SDMs) have often been developed using data sampled along elevation gradients for the forecasting of biodiversity changes, for instance to anticipate the possible ecological impacts of climate change on mountain flora [25]–[27]. These models and predictions were usually evaluated in a standard way, e.g., by comparing the predictions and observations at the level of individual species distributions, but rarely by evaluating the properties of the assemblages themselves [28]. In particular, little is known about the performance of stacked species distribution models (S-SDMs) in predicting biodiversity patterns along important ecological gradients such as elevation [29].

The S-SDM approach considers a simple stacking of individual species responses to the environment and therefore does not explicitly integrate any potential constraint on the maximum number of species that can co-occur in a given area (e.g., available energy, heterogeneity within the modelled unit, or biotic interactions; [29]). On average, it has been shown that the sum of the individual predictions tends to overestimate species richness (i.e., commission errors; [16], [21], [29]). Species assemblages can better represent environmental constraints on species richness in stressful (non-productive) environments (e.g., alpine areas) [28], [30], where diversity is primarily determined directly or indirectly by climate. In more productive environments, species responses to climatic factors alone cannot account for the key filters on the local assembly, and their stacked predictions may lead to greater species overprediction. These studies emphasise that the accuracy of prediction is not necessarily constant across geographic space or along an indirect ecological environmental gradient such as elevation.

Specific aspects of the model technique can also lead to error in species stacking that shapes biodiversity patterns along elevation gradients; some of these errors are more related to the technical aspects of modelling (e.g., the threshold for the binary classification of predicted probabilities; [20]) and others are more related to the ability of SDMs to capture the full spectrum of community assembly processes [28]. These findings support the need to better evaluate the capacity of SDMs to predict assemblage and diversity patterns and to better understand their strengths and weaknesses in doing so. However, many questions, both technical and conceptual, remain to be addressed. The related assembly hypotheses should also be tested on a large variety of ecosystems and organisms.

In particular, one remaining question is whether stacked predictions from species distribution models (S-SDMs) can reproduce existing biogeographic patterns, such as the biodiversity patterns along environmental gradients. For instance, for species richness for different clades along an elevation gradient, Rahbek [31] recognised three main patterns: (1) a monotonic reduction from the lowest to the highest elevations; (2) a hump-shaped pattern, with the maximum values at middle elevations; (3) relatively constant values from low to middle elevations, followed by a sharp decrease toward highest elevations.

In this study, our objective was to fill this gap by evaluating the ability of S-SDMs to predict known patterns of species richness for Bromeliaceae and Araceae along a wide elevation gradient in Ecuador and by comparing different ecological modelling options. We used typical herbarium species occurrence data for 142 species, as used in Elith et al. [32], combined with typical bioclimatic maps to predict potential altitudinal biodiversity patterns and compare these with actual patterns obtained from two exhaustive, independent data: (1) expert criteria extracted from bibliography [33], and data from plot transect [34]–[35]. In the central Andes, Kessler [34] identified hump-shaped curves for Bromeliaceae, whereas Araceae showed relatively constant values up to elevations of between 1000 and 1500 m, followed by a monotonic decrease. It is thus interesting to evaluate how well these patterns can be reproduced by stacking individual predictions of species distributions. More specifically, we place more emphasis on comparing the performance of the two-ensemble modelling approach [36], which combines predictions from six techniques into a single prediction of species richness. To our knowledge, such an evaluation of SDMs to reconstruct patterns of species richness along an elevation gradient has very rarely been performed [19] and never along such a wide elevation gradient in the tropics.

## Materials and Methods

### Species data

We conducted all analyses on two plant clades: the genus *Anthurium* (Araceae family; 53 species) and the family Bromeliaceae (89 species). These two groups are interesting for this purpose because (1) the taxonomic knowledge for these two plant groups is extensive and thus offers the required guarantees of reliable identification at the species level; and (2) their observed altitudinal patterns have already been thoroughly investigated [34].

We used all of the records stored in the TROPICOS database (Missouri Botanical Garden). All records and location data (latitude/longitude) were checked by specialists from the Missouri Botanical Garden (Saint Louis, USA) and Real Jardín Botánico (Madrid, Spain), and whenever possible errors (i.e., georeferencing or species identification) were corrected; otherwise, data in error were removed. As part of this data checking process, we performed a statistical analysis to detect outliers, as in Mateo [19], and analyzed the outliers individually. Outliers that presented insufficient reliability (expert criteria) were discarded.

The final occurrence maps included 17,064 point locations. The minimum sample size to obtain reliable predictions of species distribution for these dataset was determined in a previous study [37], Therefore, species with fewer than 15 occurrences (i.e., for which reliable models could not be fitted because of the small sample size) were excluded from further analyses [37], [38].

### Environmental predictors

We used the 19 bioclimatic variables (Table 1) that were available in WorldClim 1.3 [39] (http://www.worldclim.org) as predictor variables. These bioclimatic variables were derived by interpolating monthly mean temperature and rainfall data onto a digital elevation model (1×1 km grid cell). They represent biologically relevant environmental factors [40]. We did not find *a priori* reason for removing some variables and therefore kept all variables for the analyses, recognizing that overfitting may thus happen for species with low number of occurrences. We argue that such technical decision is conservative and may tend to predict more around the know presences.

**Table 1:** Environmental variables used to generate the species distribution models.

BIO1	Annual Mean Temperature
BIO2	Mean Diurnal Range (Mean of monthly (max temp - min temp))
BIO3	Isothermality (BI02/BI07) (* 100)
BIO4	Temperature Seasonality (standard deviation *100)
BIO5	Max Temperature of Warmest Month
BIO6	Min Temperature of Coldest Month
BIO7	Temperature Annual Range (BI05–BI06)
BIO8	Mean Temperature of Wettest Quarter
BIO9	Mean Temperature of Driest Quarter
BIO10	Mean Temperature of Warmest Quarter
BIO11	Mean Temperature of Coldest Quarter
BIO12	Annual Precipitation
BIO13	Precipitation of Wettest Month
BIO14	Precipitation of Driest Month
BIO15	Precipitation Seasonality (Coefficient of Variation)
BIO16	Precipitation of Wettest Quarter
BIO17	Precipitation of Driest Quarter
BIO18	Precipitation of Warmest Quarter
BIO19	Precipitation of Coldest Quarter

Bioclimatic variables are derived from the monthly temperature (units: °C * 10) and rainfall (mm). They represent annual trends, seasonality, and limiting factors.

### Species distribution modelling

We used six different techniques to model individual species distributions. Boosted regression trees (BRT) [41], generalised linear models (GLM) [42], and multivariate adaptive regression splines (MARS) [43] are group discriminative techniques that need presence/absence data. The other three techniques, i.e., genetic algorithm for rule-set prediction (GARP) [44], Gower's metric distance (GMD) [45] and maximum entropy (MAXENT) [46], require only presence data.

Absences are necessary to perform the group discriminative techniques. As herbarium collections only provide presence data, we generated pseudo-absences as similarly performed by Elith et al. [32] with the extension “Random Point Generator 1.28” (ArcView 3.2) and the following constraints [47]: (1) we generated approximately the same number of pseudo-absences as presences to avoid problems associated with unbalanced prevalence [48]; (2) to collect information on the different ecological conditions in the study area, we defined a minimum distance of 30 km between pseudo-absences [49], [50]; (3) to avoid increasing the false negative rate, we defined a buffer (30 km in diameter) around each presence from which pseudo-absences were eliminated [51], [52]. The distance of 30 km was calculated based on the information contained in the maps according to the pixel size [37].

The six modelling techniques used are standard and well described [32], [47]. We generated MARS models using MARS 2.0 (www.salford-systems.com), running 30 models per species and varying the following parameters: (1) the maximum number of basic functions; (2) whether or not interactions were allowed between basic functions; and (3) inclusion of all of the 19 WorldClim variables or elimination of the mean annual temperature and mean annual precipitation to reduce multicollinearity. We used MARS 2.0 instead of the mda library [32] related to the findings in previous works [19], [37], [53]. The GLM models were generated using BIOMOD, and quadratic terms were allowed. The MAXENT models were generated in MAXENT 2.1 with the default settings (“Auto features”, convergence = 10-5, maximum number of iterations = 500, regularisation value β = 10-4). BRUTO (BRT) models were generated in R (www.r-project.org) with the “mda” package and the parameters detailed in Elith et al. [32]. The Gower distance models (GMD) were performed in DIVA-GIS [54]. GARP Desktop 1.1.6 was used to generate 100 models per species; we selected the bottom 20% of models with the lowest extrinsic omission error, and of those, the ten models around the median of the commission error were used to generate the GARP ensemble model presented in the results section.

Each modelling technique produces models with different prediction values. The GLM generates probabilities in the range of 0–1. MARS generates scores that are not restricted to a predefined range. MAXENT generates models with values between 0 and 100. GARP generates a set of presence/absence models (in this case, we used a combination of 10 presence/absence models). The values in the GMD models have a maximum of 100, but they do not have a minimum value; these values can be used “as is” or transformed. All models were rescaled to the [0–1] range to generate ensemble models. The models were rescaled using the following equation:
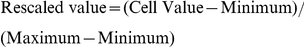



### Assessing predictive performance

The predictive performance of SDMs should ideally be evaluated with a set of independent data. In our study, this procedure was not possible for most species, due to the scant number of available collections that requires using all available data to fit the model [19]. Therefore, we assessed predictive performance of all SDMs by means of resubstitution, i.e. calculating ROC plots and the AUC statistic on the same data as used to fit the model. This is thus a different measure of model fit and some authors suggested that this can still be effective when no independent data can be left out for evaluation [26]. Although, the AUC values obtained by a resubstitution process tend to be higher than the AUC values obtained by means of evaluation [19]. In addition, we use independent data for some species of the genus Anthurium to evaluate the predictive power of models [47].

### Ensemble models

Following Araújo and New [36] and Marmion et al. [55], we calculated ensemble models for each of the species based on two different criteria: (1) ENSEMBLE-A: the average of all of the available rescaled SDMs (BRT, GARP, GLM, GMD, MARS, and MAXENT). (2) ENSEMBLE-B: the average of the four best methods (BRT, GLM, MARS and MAXENT) based on the model ranking in Elith et al. [32].

### Binary models (presence/absence)

The original models were reclassified into models of presence/absence (BINARY) using a threshold approach. Liu et al. [56] and Jiménez-Valverde and Lobo [57] present different possibilities for choosing thresholds. Because only presence data were available, we used a threshold approach that minimised the commission error [58] and allowed a maximum commission error of 0.05.

### Prediction of gamma diversity with respect to elevation

We estimated the gamma diversity in the same elevation band (500 m) used for the two ensemble models of alpha diversity. For the gamma diversity, we only used binary models and calculated the number of species predicted in at least a minimum number of pixels pertaining to each elevation band. We imposed three different thresholds by summing all of the species predicted in at least one pixel, in at least 10 pixels or in at least 50 pixels per elevation band.

### Prediction of alpha diversity along altitude

We stacked (i.e., summed) the rescaled and binarised SDM predictions for the two ensemble procedures so that we obtained four species richness maps (S-SDMs) per taxa (*Anthurium* spp. and Bromeliaceae). We estimated the altitudinal alpha diversity patterns of the four S-SDMs along the elevation gradient by calculating the mean and maximum species richness of the pixels falling into the different 500 m elevation classes.

### Evaluation of predicted altitudinal diversity patterns

To evaluate the ability of the ensemble S-SDMs to predict the alpha diversity patterns along the elevation gradient and the gamma diversity per elevation belt, we compared the model predictions to independent data compiled by Jørgensen and León-Yánez [33] for gamma diversity and to data compiled by Kessler [34], [35] for alpha diversity.

For gamma diversity, independent distributions of the 53 species considered in the genus *Anthurium* and of the 89 Bromeliaceae species were extracted from the Catalogue of Vascular Plants of Ecuador [33]. To determine whether the patterns of gamma diversity derived from the models were statistically similar to the observed patterns [59], [60], we used a reconstruction of the diversity change with elevation based on the literature. This procedure starts by generating for the most specious altitudinal band a random number between zero and the maximum number of species found for that band in the literature [33]. For the remaining altitudinal bands, we generated random numbers limited to +/−10 species from the previous altitudinal band for *Anthurium* and +/−22 species for Bromeliaceae; these two values correspond to the maximum change in the number of species found in the literature between one altitude and the next level [33]. This restriction was needed to keep the variability of the simulated values within a range similar to that of the observed values (i.e., to generate a realistic null model). We then compared the S-SDMs predicted differences in gamma diversity between each neighbouring altitudinal band with the differences generated by a set of 10,000 random simulations from our null model of altitudinal changes in gamma diversity. This comparison was based on the difference between the generated random number and the observed number of species and the average of the differences between the randomly generated and observed values across altitudinal bands. These differences were ordered, and the rank of the observed difference was divided by 10,000 to obtain the final empirical P-value. A P-value exceeding 0.05 would mean that the distribution derived from the models could have been obtained by chance alone, so that there was no association, at the 5% level, between the modelled and observed distributions.

For alpha diversity (species richness), the numerical data were not available for a similar statistical validation as performed for the gamma diversity. In this case, it was only possible to determine whether the patterns obtained with the models fit the patterns (curves comparison) established by Kessler [34], [35].

## Results

**Figure 1:**
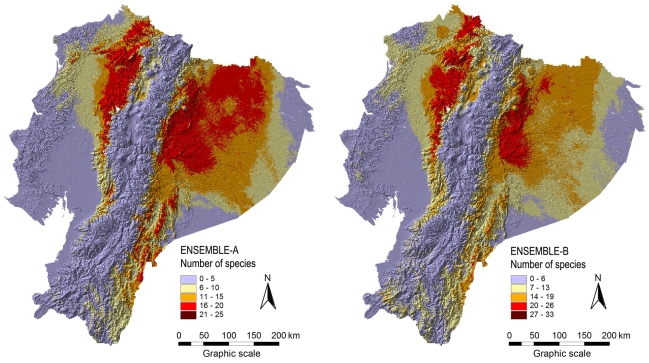
Richness (alpha diversity) of the genus *Anthurium* from the S-SDMs for the two ensemble procedures. The S-SDMs were generated by stacking the binary models of 53 species. **ENSEMBLE-A**: ensemble model of the six methods available. **ENSEMBLE-B**: ensemble model of the four best methods.

**Figure 2:**
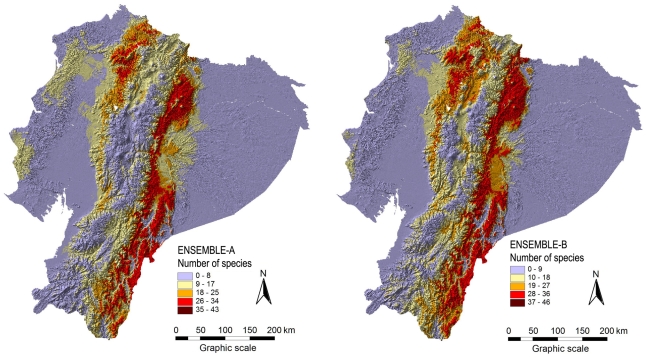
Richness (alpha diversity) of the Bromeliaceae from the S-SDMs for the two ensemble procedures. The S-SDMs were generated by stacking the binary models of 89 species. **ENSEMBLE-A**: ensemble model of the six methods available. **ENSEMBLE-B**: ensemble model of the four best methods.

All species distribution models were highly accurate in regard to AUC values that were all above 0.95 [19]. Figure 1 shows two richness models (S-SDMs) for the genus *Anthurium*, and Figure 2 shows the predicted richness for Bromeliaceae. Richness models that were derived from different ensemble procedures can be slightly different.

**Figure 3:**
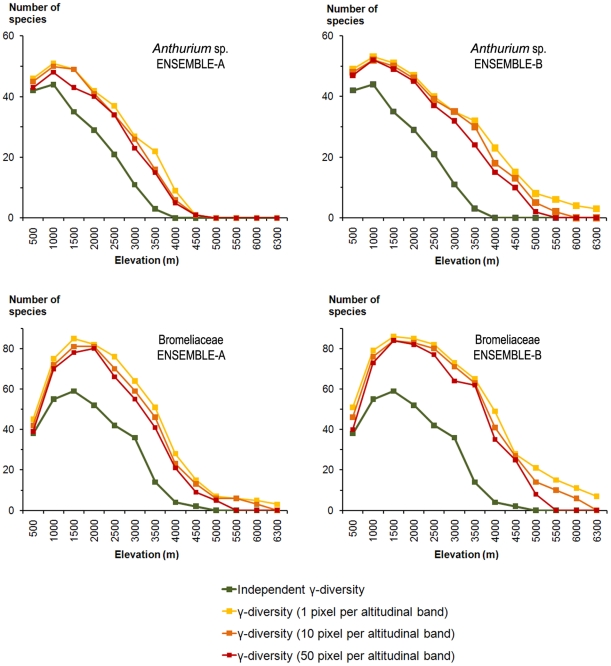
Altitudinal patterns of the potential gamma diversity in Ecuador. Altitudinal patterns for the genus *Anthurium* (above) and the Bromeliaceae family (below) according to the two ensemble modelling procedures. We imposed three different thresholds by summing all of the species predicted in at least one pixel, in at least 10 pixels or in at least 50 pixels per elevation band. **Independent γ-diversity**: the altitudinal patterns of gamma diversity in Ecuador for the genus *Anthurium* (53 species) and the Bromeliaceae family (89 species). Information from the “Catalogue of the vascular plants of Ecuador” [33]. **ENSEMBLE-A**: ensemble model of the six methods available. **ENSEMBLE-B**: ensemble model of the four best methods.

Table 2 shows the P-values of the randomisation tests, which show overall that the predicted gamma diversity patterns could be considered in most cases to be different from those derived from the null model of altitudinal changes in gamma diversity based on the literature [33] (Figure 3). However, only the ENSEMBLE-A-BINARY model could not be separated from (i.e., fits) the patterns derived from the literature.

**Table 2:** P-values of the randomisation tests.

		1 PIXEL	10 PIXEL	50 PIXEL
***Anthurium*** ** sp.**	**ENSEMBLE-A**	0.018	0.007	0.003
	**ENSEMBLE-B**	0.199	0.104	0.052
**Bromeliaceae**	**ENSEMBLE-A**	0.173	0.095	0.043
	**ENSEMBLE-B**	0.458	0.326	0.192

These tests were used to determine whether the numbers of species (gamma diversity) of two plant clades (*Anthurium* genus, Bromeliaceae family) according to the two ensemble modelling procedures in each altitudinal band were statistically different from the values derived from a null model of altitudinal changes in gamma diversity based on the literature [33]. We imposed three different thresholds by summing all of the species predicted in at least one pixel, 10 pixels or 50 pixels per elevation band. **ENSEMBLE-A**: ensemble model of the six methods available. **ENSEMBLE-B**: ensemble model of the four best methods.

There was no large difference between the three thresholds (summing all species predicted in at least one pixel, 10 pixels or 50 pixels per elevation band) used to calculate the altitudinal gamma diversity patterns, although the predicted patterns that were closest to the independent evaluation datasets were always obtained with a threshold of 50 pixels (Figure 3, Table 2).

**Figure 4:**
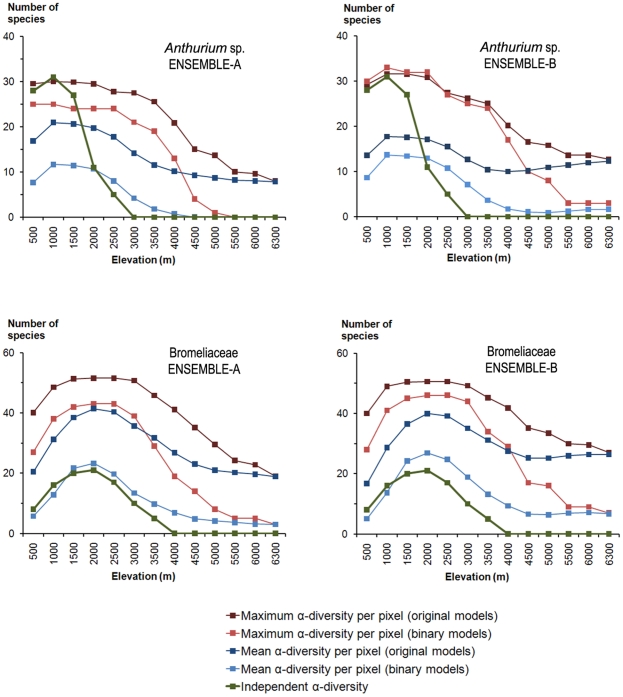
Altitudinal patterns of the potential alpha diversity in Ecuador. Altitudinal patterns for the genus *Anthurium* (above) and the Bromeliaceae family (below) according to the two ensemble modelling procedures and original predictions or binary predictions of the SDMs. **Independent α- diversity**: the Araceae and Bromeliaceae altitudinal patterns of alpha diversity modified from Kessler [35]. **Maximum value**: maximum number of species within each 500-m altitudinal belt. **Mean value**: average number of species within each 500-m altitudinal belt. **ENSEMBLE-A**: ensemble model of the six methods available. **ENSEMBLE-B**: ensemble model of the four best methods.

Figure 4 shows the predicted altitudinal patterns of alpha diversity. In both taxa (*Anthurium* spp. and Bromeliaceae), only the binary model yielded predictions that were comparable to the patterns described by Kessler [34], [35]. The original models revealed a clear tendency toward overprediction in the high elevation areas.

## Discussion

In this paper, we assessed the ability of binarised predictions of S-SDMs to reproduce patterns of species richness along a wide elevation gradient, considering both the mean alpha diversity and gamma diversity in elevation belts. We tested original rescaled and binarised predictions of S-SDMs based on two ensemble approaches for 142 plant species within two different taxonomic groups, namely the genus *Anthurium* and the family Bromeliaceae.

### Altitudinal gamma diversity patterns

All of the ensemble procedures presented a significant over-prediction of gamma diversity (i.e., high commission error; Figure 3). ENSEMBLE-A-BINARY predicted the altitudinal gamma diversity patterns with fair to good accuracy, yielding patterns similar to those derived from the literature [33]. Other models showed more serious problems of overprediction. If the goal of a study is related to conservation or to the disentanglement of spatial biodiversity patterns, the use of ENSEMBLE-BINARY or stacking results from models of that type is thus recommended. Furthermore, such an approach is also useful when the objective is to generate SDMs at the species level, for instance when associated with the conservation of a single species [61]. However, to gain generality, these findings should be confirmed with studies in other ecosystems and involving other organisms.

The absence of large differences between the three thresholds (summing all species predicted in at least one pixel, 10 pixels or 50 pixels per elevation band) used to calculate the altitudinal gamma diversity patterns was likely because many more than 1 or 30 suitable pixels are predicted for most species found in each elevation band, making the difference weak even when the threshold was 50. The patterns closest to reality were most often obtained with the threshold of 50 pixels. Indeed, these thresholds are likely to depend on the extent and resolution used for the study, and in further studies, they could be defined as percentages of the band surface area rather than absolute pixel number values.

### Altitudinal alpha diversity patterns

The first study exploring the altitudinal patterns in several plant families in the Neotropics was that of Kessler [34], who showed that Araceae had a relatively constant increase in alpha diversity values up to altitudes between 1,000–1,500 m and then decreased constantly above these elevations (Figure 4, ARAC). In our study, the richness map obtained with ENSEMBLE-A-BINARY and ENSEMBLE-B-BINARY showed altitudinal alpha diversity patterns similar overall to those in Kessler [34]. However, the Kessler data are for all Araceae species, whereas in our study only the genus *Anthurium* was considered.

For the Bromeliaceae (Figure 4, BROM), Kessler [34] found hump-shaped curves for both the epiphyte (maximum to 1,700 m) and terrestrial species (maximum to 3,000 m). The species richness predictions obtained with ENSEMBLE-A-BINARY and ENSEMBLE-B-BINARY showed the pattern most similar to that of Kessler [34] for the Bromeliaceae.

For both taxa, the results obtained by the mean model (mean alpha diversity per pixel in binary models) were closer to reality than the maximum models (maximum alpha diversity per pixel in binary models). The maximum models presented significant over-prediction of gamma diversity (i.e., high commission error; Figure 4). All of the original models also showed significant over-prediction (Figure 4). These results are consistent with previous studies dealing with alpha diversity [16], [21], [62]. The quality of the results obtained by the ENSEMBLE-A-BINARY procedure was most probably due to the binarisation procedure that was used. Indeed, the recently shown intrinsic tendency of S-SDM to overpredict species richness independently from any binarisation procedure [63] might have been balanced here by the setting of classification thresholds that minimized the commission error rates of the individual SDMs. Although this procedure appears to reduce the overprediction problem of S-SDMs, further investigation should be conducted on the effects on the assemblage composition in addition to the species richness predictions.

### Evaluation of predicted altitudinal diversity patterns

In general, studies investigating the response of biodiversity to environmental changes that are based on SDMs evaluate the accuracy of their predictions at the species level. Here we showed that highly accurate SDMs (AUC above 0.95) might lead to important discrepancies between the predicted diversity patterns from S-SDMs and the observed patterns [19]. We suggest the provision of both species-level and assemblage-level evaluation metrics when SDMs are used to investigate biodiversity patterns (S-SDMs). Robust gamma diversity estimates rarely exist; thus, such a null-model reconstruction should prove useful in further studies.

### Binary models (presence/absence)

The use of a threshold clearly improved the results (Figures 3 and 4) by decreasing the commission error rates in SDMs and thus allowing the modelled altitudinal alpha diversity patterns to better fit the observed patterns. The original models showed serious overprediction problems at high elevations that were solved when the binary models were used. As previously suggested [21], the selection of an appropriate suitability threshold can reduce error rates in both individual and ensemble SDMs, but this selection is not straightforward and the results can vary, sometimes dramatically, depending on the threshold chosen. An additional problem in the selection of reliable and stable threshold values is the lack of real absences, as in the present study. When the modelling algorithm has no information on absences, small differences in the selected threshold value can severely affect the model outputs [57]. As this threshold selection is often subjective, in this study, we chose a conservative value, which allowed a maximum commission error of 0.05. An interesting methodological line of research would thus be to study the reliability of the different thresholding approaches in species assemblage modelling, as it may help to reduce over-prediction in some cases.

### Ensemble models

Ensemble models have only relatively recently been applied to ecological modelling for predicting the spatial distributions of single species. Given the difficulty in choosing the most suitable technique [40], [64], [65], some authors suggest the combination of predictions from several modelling techniques to reduce the observed variability [36], [55], [66], [67]. In addition to improving the prediction accuracy at the species level, our results further show that ensemble modelling approaches can also allow adequate predictions when stacking multiple species distributions.

Our results show that the S-SDMs generated from all of the techniques provide better results than the S-SDMs generated from the four best modelling techniques (Table 2). Thus, in future work, research efforts may be reduced by using only techniques that are known to generate reliable, stable and accurate SDMs, without the need to select the best ones. Furthermore, in our study, both stacking strategies over-predicted species richness, particularly at high elevations, although this is likely to be study-dependent.

### Conclusions

The main conclusions that we can draw from this work are 1) Stacking ensemble models of species distributions could successfully reproduce alpha and gamma diversity patterns along a wide elevation gradient in the Andes; 2) The best results in relation to conservation or to disentangling the spatial patterns of biodiversity were obtained when stacking binary predictions of individual species distributions; 3) Stacking binary models may also reduce over-prediction, and more research must be conducted to find the most appropriate thresholding approach; and 4) The randomisation procedure used to reconstruct the gamma diversity patterns and to compare them with the S-SDM predictions represents a promising way to assess the predictions of stacked species distribution models along incompletely surveyed environmental gradients.
